# AtomNet PoseRanker: Enriching Ligand Pose Quality for Dynamic Proteins in Virtual High-Throughput Screens

**DOI:** 10.1021/acs.jcim.1c01250

**Published:** 2022-03-02

**Authors:** Kate A. Stafford, Brandon M. Anderson, Jon Sorenson, Henry van den Bedem

**Affiliations:** † Atomwise, Inc., 717 Market Street, Suite 800, San Francisco, California 94103, United States; ‡ Department of Bioengineering and Therapeutic Sciences, University of California, San Francisco, San Francisco, California 94158, United States

## Abstract

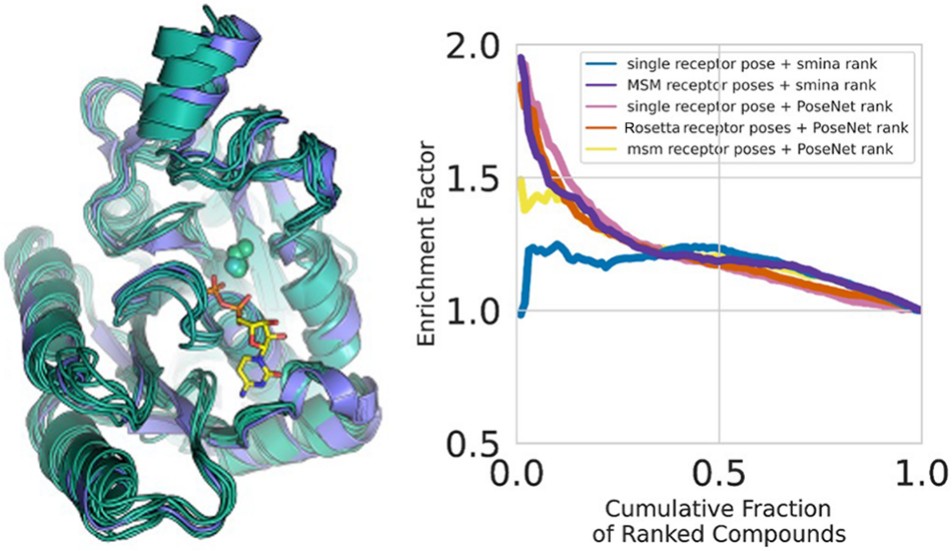

Structure-based, virtual High-Throughput Screening (vHTS) methods for predicting ligand activity in drug discovery are important when there are no or relatively few known compounds that interact with a therapeutic target of interest. State-of-the-art computational vHTS necessarily relies on effective methods for pose sampling and docking and generating an accurate affinity score from the docked poses. However, proteins are dynamic; *invivo* ligands bind to a conformational ensemble. *In silico* docking to the single conformation represented by a crystal structure can adversely affect the pose quality. Here, we introduce AtomNet PoseRanker (ANPR), a graph convolutional network trained to identify and rerank crystal-like ligand poses from a sampled ensemble of protein conformations and ligand poses. In contrast to conventional vHTS methods that incorporate receptor flexibility, a deep learning approach can internalize valid cognate and noncognate binding modes corresponding to distinct receptor conformations, thereby learning to infer and account for receptor flexibility even on single conformations. ANPR significantly enriched pose quality in docking to cognate and noncognate receptors of the PDBbind v2019 data set. Improved pose rankings that better represent experimentally observed ligand binding modes improve hit rates in vHTS campaigns and thereby advance computational drug discovery, especially for novel therapeutic targets or novel binding sites.

## 1 Introduction

Successful drug discovery campaigns rely on identifying biologically active lead molecules that are chemically distinct from known compounds for the disease target. This is especially challenging when there is little or no nearby ligand data available, as is the case with novel targets, or when novel scaffolds are distant in chemical space. Structure-based, virtual High-Throughput Screening methods are designed to overcome this challenge, by identifying novel compounds with predicted activity from vast chemical libraries, for example, MCULE^1^ or ENAMINE.^2^ vHTS is routinely applied as a first step in the drug discovery process, with hit rates surpassing those of experimental screens.^3−5^

Conventional, structure-based vHTS approaches use an empirical- or force-field-based scoring function to dock distinct ligand poses to a mostly rigid receptor and predict affinity. Underlying structure-based vHTS approaches is the assumption that receptor–ligand binding poses correlate with experimentally observed affinities. While conventional approaches have led to several exciting results, including a potent inhibitor for AmpV β-lactamase,^6^ they have several important drawbacks. First, identifying a scoring function that simultaneously gives high docking power (distinguishing correct docking poses from decoy poses) and high scoring power (generating an affinity score) has historically been challenging.^7^ For example, the widely used molecular docking scoring function AutoDock-Vina^8^ excels at pose reproduction but is less competent at correlating poses to affinity, as assessed in the CASF benchmark.^9^ Second, conventional methods bear substantial computational cost. Consequently, their ability to predict affinity is limited to small- or medium-sized chemical libraries of tens to several hundred million compounds. Third, the dynamic nature of proteins is exploited in ligand binding.^10^ Different receptor conformations can bind different ligand chemotypes, perhaps best exemplified by the “DFG-in” and “DFG-out” states occupied in different ratios by many protein kinases.^11^ Docking a ligand to a rigid receptor conformational substate that deviates from its native bound state (e.g., an apo state) can result in inaccurate predictions of the bound complex that are not useful for further drug design applications. Unfortunately, incorporating receptor flexibility and representing binding-competent receptor conformations remain challenging in conventional methods^12,13^ and substantially increase computational cost.

Machine learning (ML) and deep learning (DL) approaches can mitigate these limitations. ML can help determine features of the receptor–ligand complex that correlate with affinity to augment scoring functions and improve docking and screening power. For example, the Δ_vina_RF_20_ scoring function combines 20 ligand, protein, and pharmacophore features selected among a larger set of candidate features with random forest regression.^14^ Postscoring with Δ_vina_RF_20_ improved docking and screening (ranking) power compared to the baseline AutoDock Vina scoring function.^8^ A major advantage of learning approaches compared to conventional methods is that they can capitalize on the rapidly increasing availability of data to improve accuracy.^15^

In contrast to ML-based methods, DL-based methods avoid the requirement to specify features and instead learn relevant features directly from structural representations of the protein–ligand complex. In recent years, structure-based DL architectures^16−21^ have enabled vHTS and contributed to the discovery of numerous new leads for drugs, often for challenging protein targets and diseases.^22−24^ However, early structure-based DL approaches appeared to learn molecular features from a pose-free structure-based descriptor only, neglecting ligand binding modes and protein ligand interactions.^25^ This is especially detrimental to predicting affinity for ligands docked to noncognate receptors, which is a typical use-case in structure-based drug discovery (SBDD). To enforce sensitivity to protein–ligand interactions, more recent DL vHTS approaches include ligand binding mode information, either as a feature^26^ or as a training label in a multitask architecture.^27,28^ Simultaneous learning on ligand binding modes substantially improved activity screening and led to better generalization beyond the training set. Importantly, predicting correct ligand binding modes has merit in its own right.^29^ Precise protein–ligand interactions are vital for developing structure–activity relationships in hit-to-lead and lead-optimization applications downstream from vHTS.^30^

**Figure 1:**
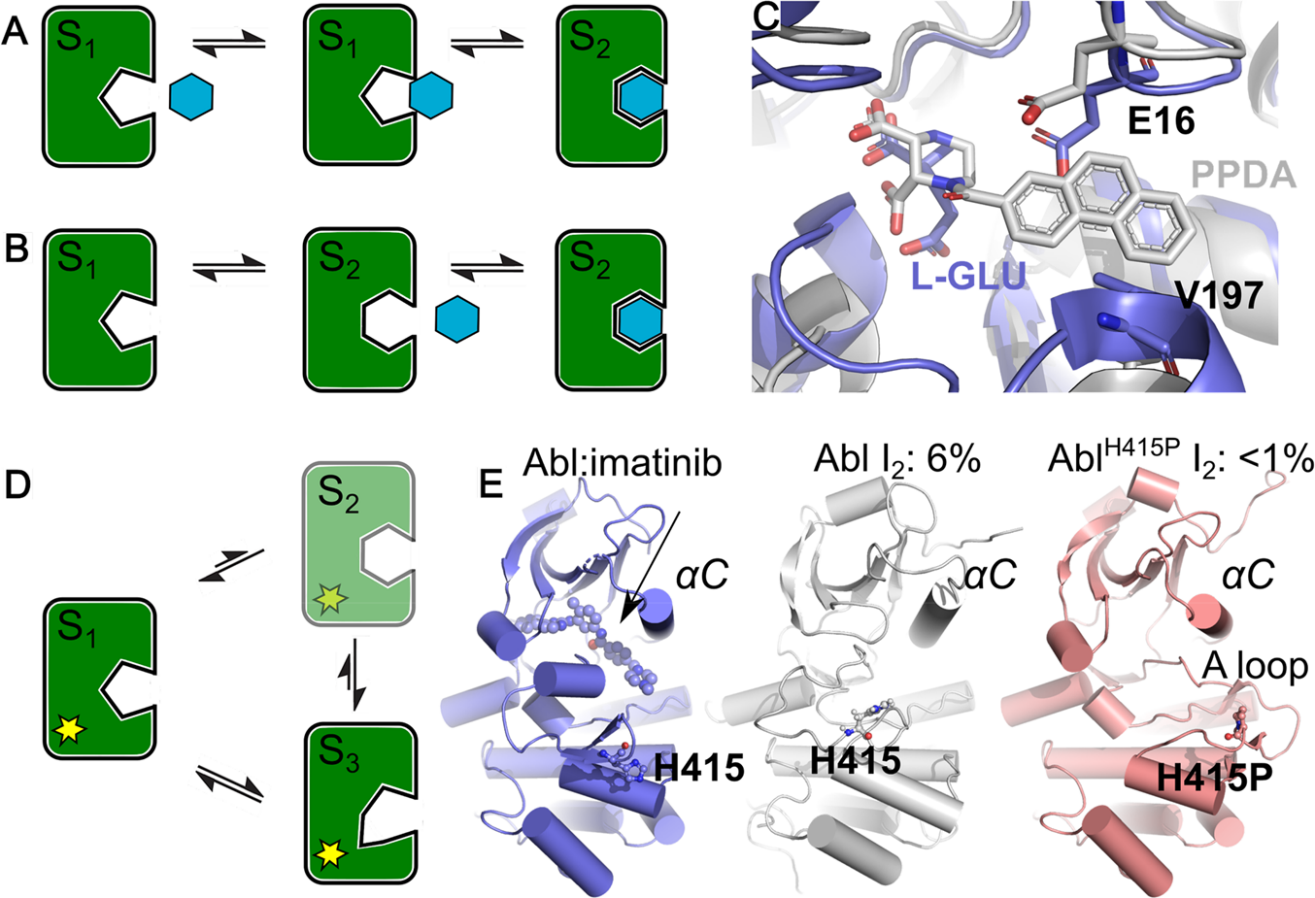
Binding site plasticity challenges vHTS in structure-based drug design. A) Induced fit. B) Conformational selection. C) The GluN2A NMDA receptor ligand-binding domain in complex with its cognate activating ligand l-glutamate (l-GLU, pdb ID 4nf8 (slate)) and antagonist 1-(phenanthrene-2-carbonyl)piperazine-2,3-dicarboxylic acid (PPDA, pdb ID 4nf6 (gray)). The l-Glu occupied site requires dramatic structural changes to accommodate the larger antagonist. D) Mutations distal to the binding site (yellow) can shift the conformational equilibrium, thereby stabilizing nonbinding substates or abrogating substates that are required for conformational selection. E) The compound imatinib in complex with Abl kinase (pdb ID 2hyy, slate). Imatinib binds Abl kinase in the inactive I_2_ state, which occupies a population of ∼6% in WT Abl (pdb ID 6xrg, gray). A distal H415P mutation in Abl kinase, at 18 Å from the active site, reduces affinity for the inhibitor imatinib 5-fold. H415P destabilizes the I_2_ state, reducing its population to below detectable levels (pdb ID 2f4j, salmon). Note the A-loop in the open conformation.

The efficacy of structure-based virtual screening campaigns relies on an adequate representation of the protein conformational ensemble. Proteins and their ligands undergo conformational exchange under physiological conditions,^31−33^ and in many cases, ligands bind through induced fit (Figure 1A)^34,35^ or bind short-lived intermediate states through conformational selection (Figure 1B).^36^ In those situations, the binding site of a crystal structure may be partially or even fully occluded in the absence of a ligand, hindering the discovery of potent binders. In cases where the protein’s cognate ligand has small molecular weight, a holo crystal structure would limit opportunities for docking larger compounds even though those could be accommodated by the protein’s full conformational ensemble (Figure 1C). Similarly, structurally uncharacterized disease mutations distal to the ligand binding site can shift the protein conformational ensemble, dramatically reducing or even depleting populations favorable for ligand binding in wild-type protein *in vivo* (Figure 1D,E).^33^ Such a situation would manifest itself by unfavorable binding kinetics in experimental assays despite highly ranked compounds in the virtual screen. Screening against multiple receptor conformations can fail to enrich experimentally validated active compounds if the conformations are higher-energy and unlikely to be accessible in solution. Careful selection of receptor states^38,39^ can mitigate these effects of protein dynamics and increase virtual screening performance.^40^

How to optimally represent protein conformational states computationally and how to generalize docking scores to (unseen) conformational substates remain important open problems. Some, but not all, proteins with ligands in the Protein Data Bank (PDB) are structurally resolved in multiple conformations. For individual systems, sophisticated and resource intensive protocols including molecular dynamics simulations and Markov state models can access biologically relevant conformations,^41,42^ but these methods scale poorly to large databases of thousands of receptors. Time-independent sampling is a less resource-intensive alternative to access conformational substates. A common limitation of screening with multiple conformations, of any origin, is the challenge in comparing docking scores for molecules docked to different conformations. This has limited the adoption of earlier ensemble approaches, but machine learning techniques can mitigate this problem.^43^ Importantly, conventional docking and scoring protocols cannot internalize receptor conformational variability encoded in the thousands of structurally resolved receptor–ligand complexes in, for example, the PDBbind data set.^44,45^ While DL approaches are relatively underexplored in ensemble docking, they can, in principle, learn and generalize how receptor flexibility accommodates distinct binding modes, both of cognate and noncognate ligands.

Here, we introduce a DL-based method for binding mode prediction that exploits protein conformational ensembles instead of single structures in vHTS applications. Starting from a protein crystal structure or homology model, we use Rosetta’s comparative modeling protocol (RosettaCM)^46^ to sample low-energy conformations near the crystal structure to generate a six-member conformational ensemble, which includes the starting structure. We applied this protocol to each receptor-compound pair in PDBbind v2019 to generate FlexPDBbind v2019, a curated data set of conformational ensembles of drug targets. To enrich the quality of compound poses docked to these ensembles, we adapted AtomNet GRAPHite, a new directional message passing graph convolutional network, for Pose Ranking applications, which we describe as AtomNet PoseRanker (ANPR). To maximize learning on distinct receptor conformations, we additionally trained ANPR on noncognate poses, obtained from cross-docking ligands to distinct crystal structures of the same protein (Uniprot identifier) in the PDBBbind v2019 data set.

We first demonstrate that ANPR rankings enrich pose quality for compounds docked to single conformations of their cognate receptors as well as noncognate receptors in the cross-docked PDBbind v2019 data set compared to conventional approaches. Our results indicate that training on a cross-docked data set teaches ANPR to recognize distinct poses as valid in different receptor conformations. ANPR trained on FlexPDBbind v2019 achieved nearly the same enrichment, suggesting that computationally sampled conformational ensembles can augment experimental ensembles when the latter are not available. Finally, we examined how conformationally diverse ensembles affect enrichment of active compounds in a virtual screen of Abl kinase. Ensemble-based procedures generally outperformed those using a single conformation. Strikingly, however, we found that using a *single* conformation with a cross-docked trained ANPR achieved nearly identical enrichment as that using an ensemble, suggesting that DL approaches can learn and infer receptor flexibility from the training set.

## 2 Methods

### 2.1 Preparing Train and Test Data Sets

We downloaded the PDBbind data set v2019 (http://www.pdbbind.org.cn) and removed entries that contained cofactors, incomplete ligands, more than one ligand, incorrect valences, entries annotated as “NMR”, or entries for which the ligand was annotated as a peptide. This resulted in a data set containing 4,593 crystal structures from the “refined” set and 10,011 structures from the “general” set. We split the data set into train (10,919) and test (5,206) sets by requiring that receptors share less than 70% or 50% sequence similarity between the sets (“seqsim70” and “seqsim50” splits). For comparison, we also generated train and test sets identical in size to the seqsim70 and seqsim50 sets but split by enforcing that these sets do not share identical Uniprot identifiers (“Uniprot split”).

### 2.2 Preparing Ligand Structures

We generated ligand structures for docking based on the ligands provided by PDBbind. To avoid biasing poses toward the crystal structures, and in contrast to previously reported studies, we discarded the native compound conformation and generated a UFF energy-minimized starting conformation from the ligand SMILES supplied by the PDB using RDKit.^47^ This approach better reflects commercial vHTS campaigns, where a crystal pose or experimental structure of the ligand is typically lacking. We used a single low-energy ligand conformation for docking.

### 2.3 Preparing Receptor Structures

**Figure 2:**
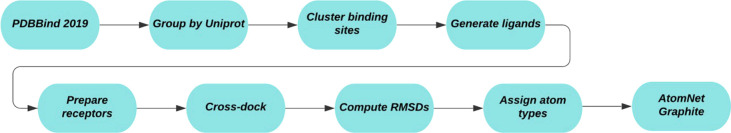
Flowchart illustrating data processing pipeline. Beginning from the PDBbind v2019 protein–ligand data set, we clustered ligands for all structures of the same Uniprot into distinct binding sites to define cross-docking groups. We used Rosetta (weekly release r231, 2019) to prepare the structures for docking, including filling in missing side-chain atoms and adding protons. We prepared 3D conformations for each ligand using RDKit and used these conformations to dock to the crystal structures within each binding site cluster. We additionally prepared conformational ensembles using the Rosetta hybridization protocol and docked ligands within each binding site cluster.

We prepared the receptors for docking by a three-step process. For consistency with our generated ensembles, we first read the crystal structure into Rosetta and removed any crystallographic buffer or water molecules but retained metal ions of the following types: Na, Fe, Mg, K, Mn, Zn, and Ca. Using Rosetta, we filled in missing atoms for incomplete protein residues. We then define a bounding box surrounding each initial ligand used as the search space for subsequent docking. For this study, we accepted the protonation states of titratable residues found in PDBbind (Figure 2).

### 2.4 Cross-Docking

We superimposed receptors of the same Uniprot ID using the chain identifiers present in the “pocket” pdb file from PDBbind, keeping receptors that superimposed to within 5 Å. We identified different binding sites within the same Uniprot ID by clustering the center of mass of the superimposed ligands with DBSCAN^48^ (parameters eps = 5., min_samples = 1). A small number of Uniprot IDs (“targets”) are represented by hundreds of receptor-compound pairs in PDBbind. To avoid those from dominating the cross-docked data set, we randomly selected five representatives from among the receptor-compound pairs to be included in the cross-docking procedure. Cross-docking was performed on all members of each binding site cluster. The final training set consisted of targets that sampled at least one low-RMSD pose. This procedure gave a total of 27,166 target-compound pairs.

### 2.5 Ensemble Docking

We generated five diverse receptor conformations using the Rosetta hybridize protocol (Supporting Information) with the score3 and score4_smooth_cart scoring functions in stages 1 and 2 of the centroid stage and the ref2015_cart scoring function with metalbinding_constraint in the full atom stage. Ligand parametrization relied on Rosetta’s templates whenever available or was generated with Rosetta’s molfile_to_params.py script using default settings. We used default weights for intraligand hetatm_cst_weight = 1.0 and receptor–ligand hetatm_to_protein_cst_weight = 1.0 constraints. Fragment insertions were disabled in stage 2: fragprob_stage2 = 0.0. We adjusted the stage 2 Monte Carlo temperature to 0.5: stage2_temperature = 0.5. Next, we subjected each hybridized receptor conformation and the crystal structure to six rounds of energy relaxation with Rosetta’s FastRelax protocol, retaining metal coordination with the SetupMetalsMover protocol combined with a metalbinding_constraint = 1.0. We observed that protein side-chain metal coordination was not preserved consistently throughout the hybridize protocol. In some cases, that led to final models with unsatisfied coordinate-covalent bonds. We created a final ensemble of six receptor conformations by selecting the conformation with the lowest Rosetta energy from each of the six relaxed conformations starting from the hybridized models or crystal structure.

### 2.6 Docking

We used a slightly modified version of the smina docking software^49^ with the vina scoring function to generate binding poses for the receptor-compound pairs with command line parameters --exhaustiveness 384 --energy_range 99999 --num_modes 64 --mc_steps 3 --minimize_iters 40 --accurate_line --approximation linear --autobox_add 2.0 --seed 42. Our modification introduces the additional parameter mc_steps which is used to tune the number of steps in the Monte Carlo search. For cross-docking, we determined bounding boxes for the docking site using the largest ligand in the target class. We then added 2 Å to define a small buffer region surrounding this expanded search space (using the --autobox_add parameter). The remaining smina input parameters were optimized primarily for computational efficiency in docking ultralarge data sets, for higher rates of successful pose generation even at lower ranks, and to ensure a diverse set of negative examples. For each receptor-compound pair, we generated up to 64 poses with the default 1 Å minimum difference between poses. We labeled poses within 2.5 Å of the native pose a “hit”, and those greater than 4 Å a “miss”. Although pose classification work often uses a 2.0 Å threshold to define success,^9^ we selected the slightly more generous 2.5 Å threshold to accommodate slight differences in alignment within our sampled ensembles, a common approach when representing receptor conformational flexibility.^50^ Poses in between were discarded from the training set but retained in the testing sets. Note that this process results in a highly imbalanced data set; for cross-docking, the data set consists of 5.0% positive examples, 15.5% intermediate examples, and 79.5% negative examples.

We calculated the RMSD between corresponding heavy atoms of docked poses and native crystal structures of the corresponding compounds by first matching substructures using RDKit and accounting for symmetric substructures by using the minimum RMSD in the case of multiple matches. For the final data set, we required that each compound adopted at least one pose within 2.5 Å of the native pose, evaluated after superposition of protein structures in the case of cross-docking.

### 2.7 Descriptor Generation

From each docked complex, we generated descriptors for use in training and testing our models. We describe each heavy atom with its corresponding SYBYL atom type indicating its chemical environment, generated by converting to Mol2 format using OpenBabel.^51^ We do not represent protons explicitly in our final descriptor sets, and we treat all metals as a single type. We treat the protein and ligand as distinct entities and encode their atom types separately.

### 2.8 Markov State Models of Abl

We obtained structures for 16 Abl kinase macrostates from the manuscript by Roux and co-workers (PDB ID 2HYY).^52^ We prepared a matching 16-member conformational ensemble using our Rosetta-based ensemble-docking protocol. For each of those ensembles, we prepared the receptors for docking following our standard protocol described above. We prepared the single receptor conformation for this analysis based on the crystal structure 2HYY. We selected 8,946 compounds from our internal databases to dock to Abl kinase, including molecules with known activity (3,205), known nonbinders (4,459), and random molecules (1,282). We ranked compounds using the ANPR model trained on a data set that excluded Abl kinase or receptors with more than 70% sequence similarity to Abl kinase. We calculated enrichment factors as ef(ν) = (*ap*)^–1^*a*(ν)/*n*(ν), where *ap* is the total fraction of actives in the data set, *a*(ν) is the number of actives in the top ν-percentile, and *n*(ν) is the total number of compounds in the top ν-percentile.

### 2.9 AtomNet PoseRanker

**Figure 3:**
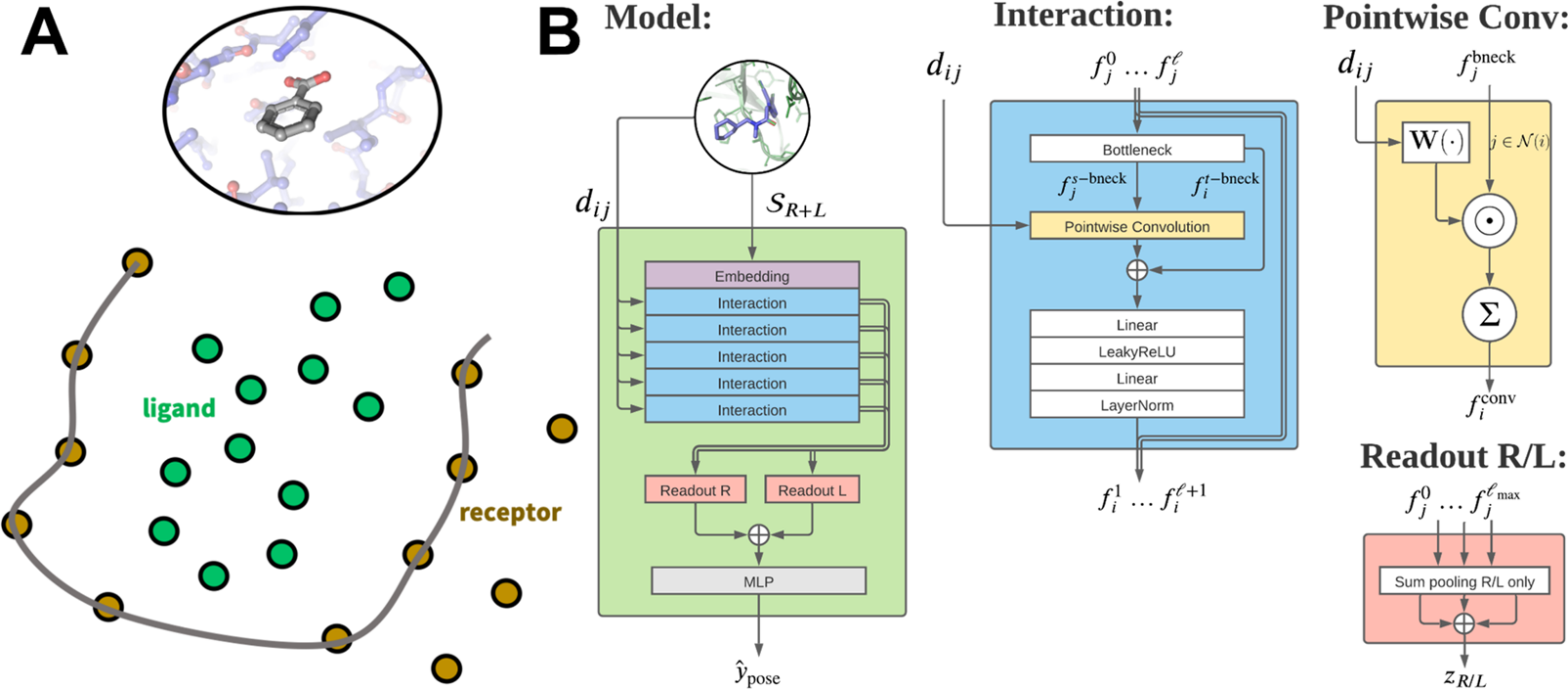
Architecture of AtomNet GRAPHite, a directional Message Passing Neural Network. A) Ligand and receptor atoms are represented as nodes in AtomNet Graphite, omitting their covalent structure. B) Overview of AtomNet GRAPHite’s graph convolutional network architecture. After an initial embedding, any number of interaction blocks can be stacked. Each interaction block features a learnable pointwise convolution and can be configured to allow interactions between ligand and/or receptor atoms with a customizable layerwise cutoff. A final readout layer extracts relevant features from ligand and receptor embeddings independently and passes them through a final multilayer perceptron.

Underlying ANPR is our GRAPHite network architecture, a directional Graph Convolutional Network (GCN)^53,54^ in which nodes encode receptor or ligand atoms (Figure 3A). We do not impose a covalent structure on the graph. Instead, pairs of atoms at layer  within a radial threshold  of each other can pass messages along a directed edge. To enable a focus on the ligand–receptor interface, we use a flexible framework that allows us to configure distinct sets of “source” () and “target” () atoms for messages in each layer (Figure 3B). At each layer, we can select a new set of target nodes from ligand ( = ), receptor (), or complex ( =  = ) nodes. The target nodes of the previous layer  will be selected as the source nodes .

We define a feature vector  of  features for source atoms , with *f*_*i*_^0^ initialized using a one-hot encoding of SYBYL atom types. The combination of the radial threshold  and layer-wise choice of target nodes gives a layer-wise neighborhood , where *d*_*ij*_ = ||**r**_*i*_ – **r**_*j*_|| is the pairwise distance between atoms *i* and *j*. Our interaction block (Figure 3B) is based upon a continuous filter convolution^54^
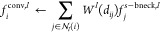
where  is a bottleneck source feature constructed by concatenating all layers of matching source atoms . This bottleneck allows us to change the number of filters on a layer and avoid mismatched filter sizes.

We use convolutional kernels  constructed from linear combinations of zero^th^-order spherical Bessel functions with a layer-dependent normalization *j*_0_(*x*) = √(2/*R_c_^l^*) sin *x*/*x*. Here, *z*_0*n*_ is the *n*^th^ zero of *j*_0_(*x*), and  are learnable weights, and the number of basis functions is chosen as , where . This parametrization ensures that the convolutional kernels vanish at the edge of an internal neighborhood. Note that since we are not calculating forces, we do not need a smooth cutoff at the boundary. After the graph convolution step, we add a skip connection based upon the target-bottleneck  and apply a Linear – LeakyReLU – Linear – LayerNorm multilayer perceptron (MLP). This bottleneck layer is used instead of the conventional residual connection at the output of a convolution block. We found empirically that this performed better than standard skip connections from the previous layer of the same set of source atoms.

To construct our final embedding, we concatenate readout operations to the ligand and receptor features independently at each layer:  and , where  and  are respectively the ligand and receptor-only pooled features at layer  (Figure 3B). Note that if no features are included at a layer, we do not include them in the embeddings. Finally, we concatenate all these embeddings together, *z*_read_ = *z*_*L*_ ⊕ *z*_*R*_, and apply a Linear – LeakyReLU – Linear MLP to obtain the ANPR ranking logit. We listed all hyperparameters in Table S1.

## 3 Results

### 3.1 Ligand:Receptor Interfaces Contribute to Pose Prediction Accuracy

Several studies suggest that structure-based vHTS CNN-based methods minimally rely on features of the receptor^55,56^ but instead distinguish active from inactive compounds primarily by ligand-based features. Carefully designed data-augmentation can draw in receptor-based features in these models, thereby promoting generalizability.^57^ By contrast, receptor features in graph-based models can affect binding mode prediction.^58^ These reports of “memorization” in bioactivity prediction, which can also affect other AtomNet applications,^59^ motivated us to design AtomNet’s configurable convolutional layers to deconvolve the role of the receptor and the ligand in predictive performance and to probe and optimize the role of ligand:receptor interactions. Note that in the application to pose quality studied here, ligand 1D features are identical in positive and negative examples, although 3D conformation and receptor interactions vary. A hyperparameter controls the number and configurations of AtomNet’s message passing layers. If ‘l’ denotes the set of ligand atoms, ‘r’ denotes the set of receptor atoms, and ‘lr’ denotes the combined set of atoms; each layer can pass messages from ‘l’ to ‘r’, ‘r’ to ‘l’, ‘l’ to ‘l’, and ‘r’ to ‘r’, and each ‘l’ or ‘r’ in these can be replaced by ‘lr’.

To test the role of the ligand:receptor interface in classifying poses, we evaluated the effect of distinct layer configurations on distinguishing correctly docked poses from incorrectly docked poses for the Uniprot split PDBbind data set. We tested layer configurations featuring transitions between ligand and receptor layers to focus on interface features. Table 1 reveals that, while the network demonstrates some capacity to identify correct poses based on ligand features alone, the test AUC (PDBbind self-docking, Uniprot split) increased dramatically when we included the ligand:receptor interface in the layer configurations (test AUC > 0.9), compared to a layer configuration between ligand atoms only (test AUC = 0.67). When we probed the interface with additional convolutional layers, we observed a peak in test AUC at 0.92. Adding more layers did not result in further improvements. These findings suggest that our graph convolutional architecture is sensitive to mechanisms of molecular recognition in structure-based drug design. To minimize the potential of ligand- or receptor-based pattern memorization, for the remainder of the study we adopted the “l→l→r→l→r→l” layer architecture that includes edges only *between* ligand–receptor atom pairs.

**Table 1:** Distinct Configurations of Convolutional Layers Respond Differentially to Mechanisms of Molecular Recognition^a^

layer configuration	test ROC AUC
l→l→l→l→l→l	0.67
lr→lr→lr→lr→lr→lr	0.91
l→l→r→l→r→l	0.91
l→l→r→l→r→l→r→l	0.92

a Test AUC for distinct convolutional layer configurations in ANPR. Test AUCs were computed from classifying poses obtained from self-docking on the PDBbind data set Uniprot split.

### 3.2 AtomNet PoseRanker Improves (sm)vina and ML-Based Rankings

Next, we compared ANPR’s ability to classify docked poses on the PDBbind data set to similar classifiers. On the Uniprot split, ANPR’s test AUC was 0.93. Other methods similarly reported AUCs in the 0.86–0.94 range^27^ (Table 2). By training simultaneously on poses and activity, Lim et al.^28^ achieved an AUC = 0.94 on their data set. Direct comparison of AUCs with these methods is difficult owing to differences in the way the data is split which can lead to memorization effects^27^ and slight random initializations of the methods.

**Table 2:** Pose Classification Compared to Other Methods Across Different Train/Test Splits^a^

	ROC AUC (PR AUC)		
method	Uniprot	seqsim70	seqsim50
ANPR	0.93 (0.47)	0.90 (0.45)	0.89 (0.39)
smina docking	0.82	0.82	0.81
Cornell et al.^58^	0.86		
Ragoza et al.^60^	0.815^c^		
Lim et al.^28^	0.94^b^		

a Baseline random performance is 0.5 for ROC AUC and 0.05 for PR AUC, reflecting the presence of 5% true positive good poses in the test set.

b In contrast to other methods in this table, the model of Lim et al. was trained simultaneously on poses and activity.

c Three-fold cross-validation on 90% sequence identity.

We then tested ANPR’s ability to generalize out of the training data by evaluating its performance on the seqsim70 and seqsim50 data splits, which challenge memorization effects of the training data. We observed a small decrease in performance from AUC = 0.93 to 0.89.

**Figure 4:**
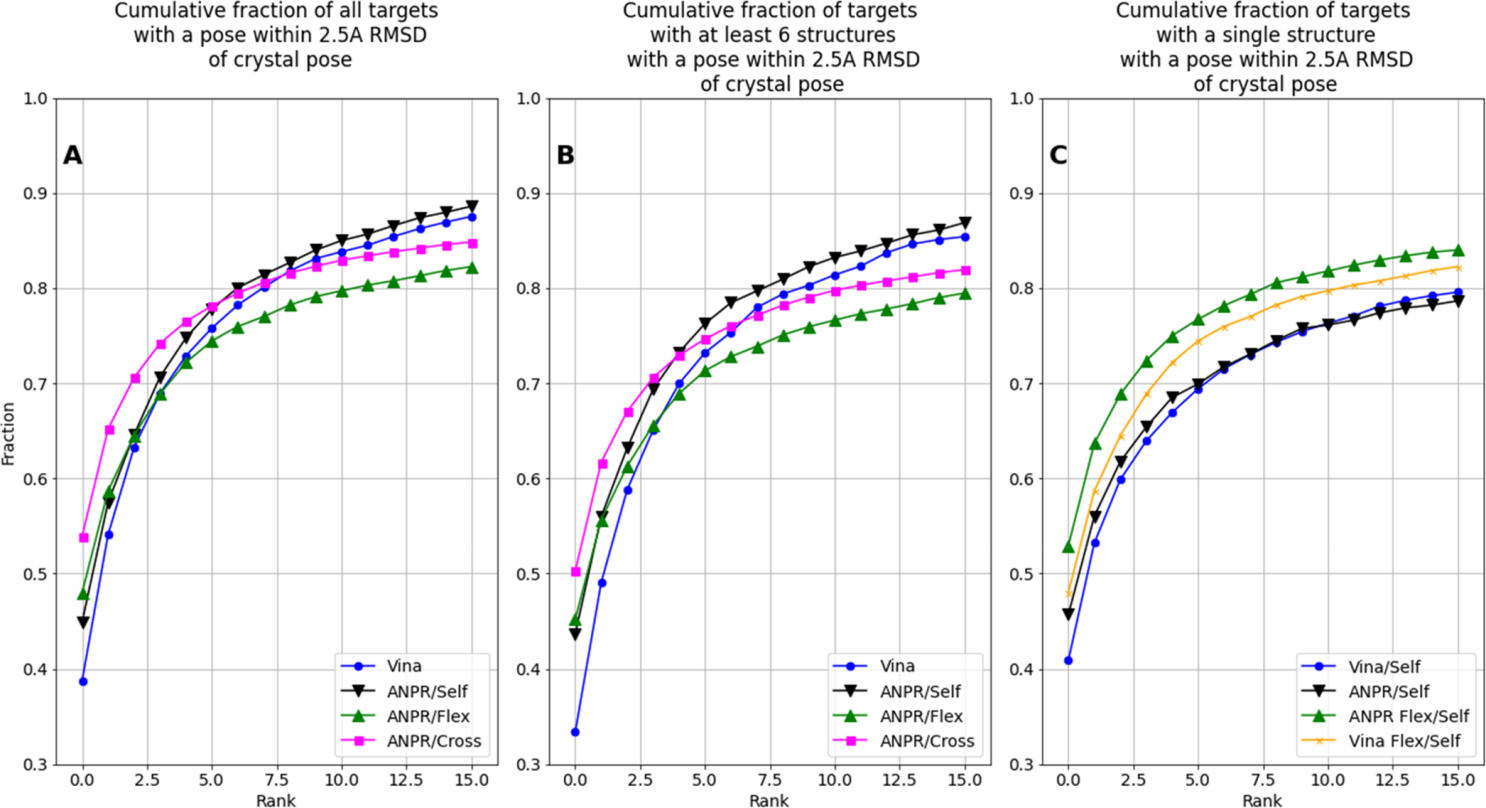
Comparison of pose ranking performances. Cumulative fraction of targets with docked poses within 2.5 Å RMSD of the crystal pose across all receptors within a target class by pose rank for the seqsim50 split according to vina (blue), ANPR trained on self-docked crystal structures (black), ANPR trained on cross-docked crystal structures (pink), and ANPR trained on sampled conformational ensembles (green). Each figure shows the top 16 ranked poses for each docking attempt in A) the full data set and B) targets that include at least six PDB structures, for a direct comparison of experimental and sampled ensembles. C) Models applied to a data set of single receptor conformations for all targets for which only one crystal structure is available. For reference, the vina ranking for the same targets across a six-member sampled ensemble is shown in orange.

In practical applications, pose ranking within a particular target class is more important than the pose quality across all targets as reported by AUC. ANPR significantly enriched the fraction of poses within 2.5 Å of the native pose among the top-*n* ranked poses compared to smina docking (Figure 4). When “gap” poses between 2.5 and 4 Å were excluded in training, 45% of the top poses in ANPR were within 2.5 Å of a crystal pose by target class (Figure 4A black line), compared to 39% of the top smina poses on the (most challenging) seqsim50 test set including “gap” poses (Figure 4A, blue line).

### 3.3 AtomNet PoseRanker Enriches Intratarget Compound Rankings in Cross-Docking

While often the redocking performance of a deep learning model is reported to evaluate its performance, correctly predicting the binding mode of a new ligand, possibly to a new target, is more important in SBDD. The new target may even lack a crystal structure, so vHTS will need to rely on a closely related structural model or a homology model. We therefore trained and evaluated ANPR on a cross-docked data set (Methods). Table 3 reports the AUC for ANPR compared to smina docking.

**Table 3:** Cross-Docking Pose Ranking/Classification Compared to smina Docking^a^

	ROC AUC (PR AUC)		
method	Uniprot	seqsim70	seqsim50
ANPR	0.91 (0.46)	0.90 (0.45)	0.86 (0.36)
smina docking	0.78	0.79	0.78

a Baseline random performance is 0.5 for ROC AUC and 0.05 for PR AUC, reflecting the presence of 5% true positive good poses in the test set.

Unsurprisingly, distinguishing favorable docking poses from unfavorable poses is more challenging in cross-docking compared to self-docking, reflected by reduced AUCs for ANPR and smina docking. There are numerous challenges in cross-docking: 1) the binding site often has a different size and/or shape owing to different (equilibrium) backbone and side-chain positions; 2) amino acid insertions, deletions, or substitutions can alter the steric and electrostatics characteristics of the site; 3) changes in solvation characteristics; and 4) experimental conditions like crystal packing, etc. Accordingly, test AUC were reduced slightly compared to self-docking across all data splits (Table 3).

Despite its reduced ROC-AUC, we observed marked intratarget early enrichment among the top-ranked poses of the model trained on cross-docked crystal structures compared to the model trained on cognate receptors (Figure 4A, top-1 54% (pink) vs 45% (black)). Our highly imbalanced data set could mask true model performance of the cognate model reported by ROC-AUC (0.89 vs 0.86 for the cross-docked model). However, we observed a concomitantly slightly elevated PR-AUC for the cognate model (0.39 vs 0.36) suggesting both models detect positives across poses at similar rates. Nonetheless, the cross-docked model appears more confident about good poses, likely because similar good poses occurred across multiple receptor conformations, whereas invalid poses were reproduced less across receptors. We note that our baseline smina performance (blue) is lower than similar applications using PDBBind,^61^ likely due to differences in docking setup and parameters; for our intended application in reranking, we optimized for sampling depth rather than top1 performance.

## 4 Ensemble-Based Docking

Next, to further examine how learning receptor conformational diversity can help ANPR recognize valid binding modes, we developed a protocol that creates an ensemble of computationally sampled receptor conformations for docking based on Rosetta’s comparative modeling protocol^46^ (Methods). Aside from providing conformational variability when multiple experimental structures are lacking, computationally sampled ensembles offer advantages over those constructed from experimental structures for a pose training task, including removing variations due to mutations or differences in construct, normalizing the numbers and sources of different conformations available for each protein in the data set, and permitting the trained model to be used in practical applications to docked poses to homology models produced through Rosetta-based pipelines.

### 4.1 FlexPDBbind: Ensemble Models for 13,000 Biomolecular Complexes

**Figure 5:**
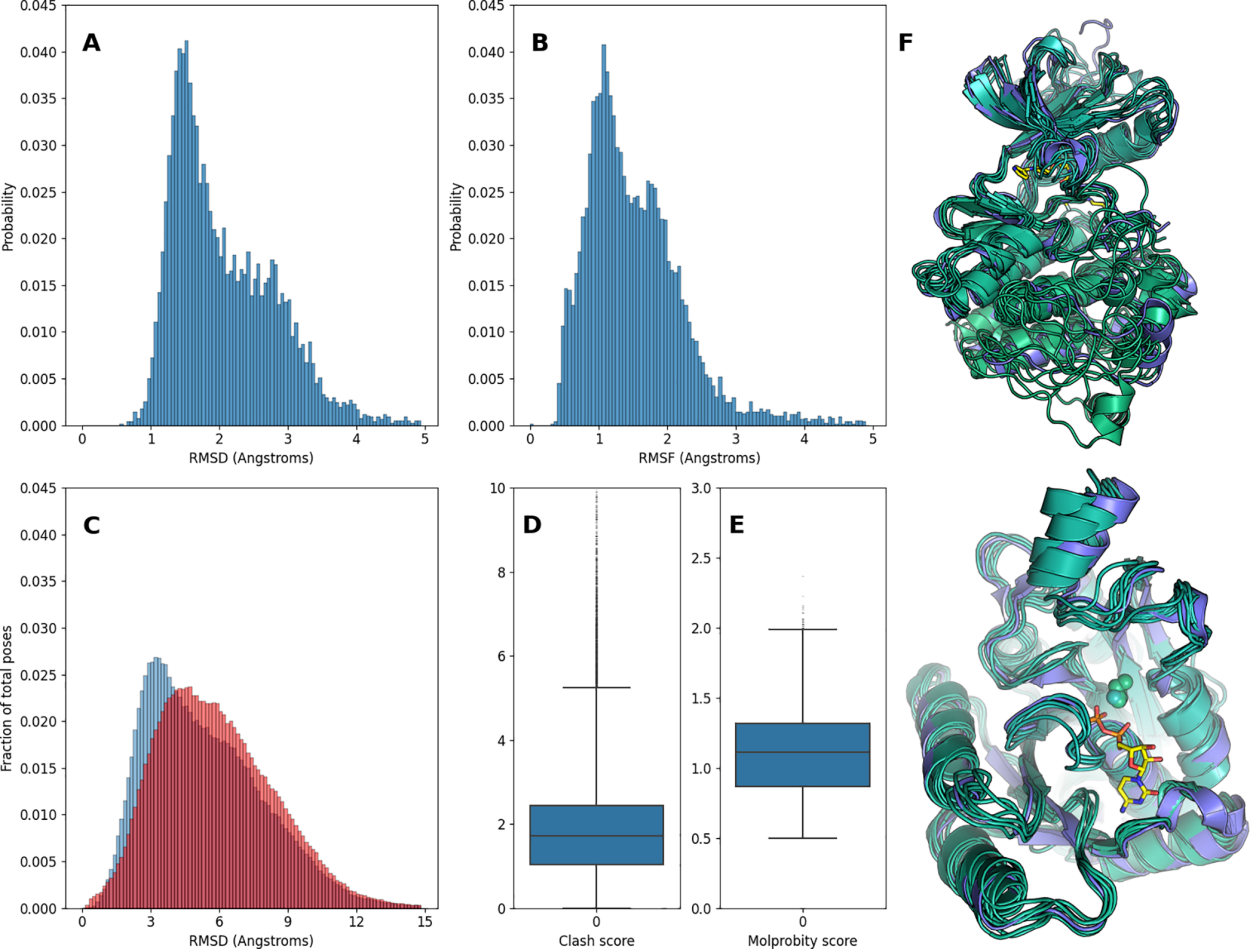
Descriptive statistics of FlexPDBbind ensembles. A) RMSD and B) RMSF distributions for six-member sampled ensembles. C) Distribution of RMSDs of ligand poses docked to sampled ensembles (blue) and crystal structures (red). D) Molprobity clash scores and E) overall scores for sampled ensembles. F) Examples of a sampled conformational ensemble for ABL1 in complex with an inhibitor (left, PDB ID5HU9) and dethiobiotin synthetase in complex with CDP (right, PDB ID6CVF) with the crystal structure in purple and ensemble members in green. The ligand corresponding to the crystal structure is shown in yellow.

For each biomolecular complex in the PDBbind “refined” and “general” sets, we generated a six-member conformational ensemble. We applied Rosetta’s FastRelax protocol to the crystal structures to generate the first member and then repeatedly applied the Hybridize and FastRelax protocols to generate up to five additional conformations (Methods). This resulted in 13,063 biomolecular ensembles, for a total of 71,825 conformations. The mean Root Mean Square Deviation (RMSD) of the ensembles calculated over the CA atoms is 1.57 Å, and the Root Mean Square Fluctuation is 2.10 Å (Figure 5A,B). This compares to a mean RMSD of 1.24 Å for the structurally aligned crystal structures. To evaluate the structural quality of the generated models, we calculated clash scores and overall Molprobity scores with Molprobity^62^ (Figure 5D,E). We discarded ensembles with outlying clash scores (scores over 10). We then docked ligands to each conformation of their cognate receptor using the protocol detailed in Methods. The RMSDs of docked ligand poses calculated to their starting pose in the source receptor conformation reveal a distribution sharply peaked around 3.5 Å with a long tail, illustrating enrichment of low-RMSD poses in sampled ensembles (Figure 5C). The distribution broadens when the RMSD is calculated with respect to the pose from the crystal structure, owing to random offsets in rigid body transformations of the generated structures (Figure 5C, red). Figure 5F shows two examples of conformations in FlexPDBbind.

### 4.2 Ensemble-Docking Mitigates Limitations of Induced-Fit Docking

ANPR trained on the FlexPDBbind ensemble of cognate receptors slightly improved top-1 enrichment compared to the model trained on single conformations (Figure 4A; top poses: 48% (green) vs 45% (black) for single conformations). However, we did not observe improved enrichment beyond the top poses. Unsurprisingly, the model trained on cross-docked crystal structures outperformed the FlexPDBbind-trained model (Figure 4A pink; top poses: 54%), likely owing to receptor conformations that are closer to physiological substates and to a more diverse representation of binding modes for targets with a large number of receptor-compound pairs in PDBbind. Approximately 80% of targets in PDBbind have fewer than six structural representations. For a more direct comparison between the cross-docked set and FlexPDBbind, we repeated the analysis limited to targets that have at least six structural representations in the cross-docked set (Figure 4B). While the top-1 enrichment on the FlexPDBbind subset was similar to that of the full FlexPDBbind set (Figure 4A), somewhat surprisingly the enrichment *gain* between the cross-docked and FlexPDBbind was substantially reduced on these subsets.

Strikingly, when we applied an ANPR model trained on sampled ensembles to predict binding modes using a data set of single receptor structures, we found that top poses were enriched over the model trained on single structures (Figure 4C, green vs black line). Notably, ANPR enrichment on *single* conformations exceeded that based on vina rank across six-member sampled ensembles (Figure 4C, green vs orange line). While, again, the improved ANPR performance could in part perhaps be attributable to the larger training set for the sampled ensembles, these results do suggest that the model retains information about flexible binding sites, illustrating the value of performing additional conformational sampling where experimental data is limited.

### 4.3 Ensemble-Trained AtomNet PoseRanker Enriched Abl Kinase Actives on a Conformational Ensemble and a Single Conformation

#### 4.3.1 Abl Kinase

As a practical application, we examined the effects of receptor conformational ensembles and compound pose reranking in identifying active compounds of Abl kinase (Abelson tyrosine kinase). Abl is of clinical significance due to its causal role in chronic myelogenous leukemia (CML); in about 90% of cases of CML, a chromosomal translocation forms a “Philadelphia chromosome”, which creates a fusion between the Abl and break-point cluster (Bcr) genes to produce a protein with constitutive kinase activity.^63,64^ Inhibitors of Abl kinase activity are in clinical use as treatments for CML and other cancers since the US approval of the targeted inhibitor imatinib in 2001 and have significantly improved clinical outcomes.^64^

Kinase inhibitors are often divided into multiple classes or types depending on their binding mode.^65^ Type I inhibitors are considered to bind to the “DFG-in” protein conformation, while type II inhibitors bind the “DFG-out” conformation. The DFG-in conformation is sterically incompatible with the most common binding mode of type II inhibitors. Among known mutations conferring resistance to imatinib and related compounds, many exert their effect by changing the protein’s conformational distribution and reducing occupancy of the favored binding state (Figure 1E). As a result, identifying both type I and type II inhibitors in a structure-based virtual screen of the ATP binding site is important for identification of new molecules of potential clinical utility and requires the use of multiple protein conformations to adequately sample native-like ligand binding modes.

We evaluated the power of our ensemble-docking protocol to enrich known Abl inhibitors over an in-house curated library of negative examples including known nonbinders and randomly selected molecules. We compared a conformational ensemble (*n* = 16, RMSF = 2.7 Å) generated using our protocol to a conformational ensemble (*n* = 16, RMSF = 2.4 Å) derived from a Markov state model (MSM) based on extensive molecular dynamics simulations.^42,52^ We also calculated the enrichment factor from the smina rank of docked poses to the (relaxed) crystal structure.

**Figure 6:**
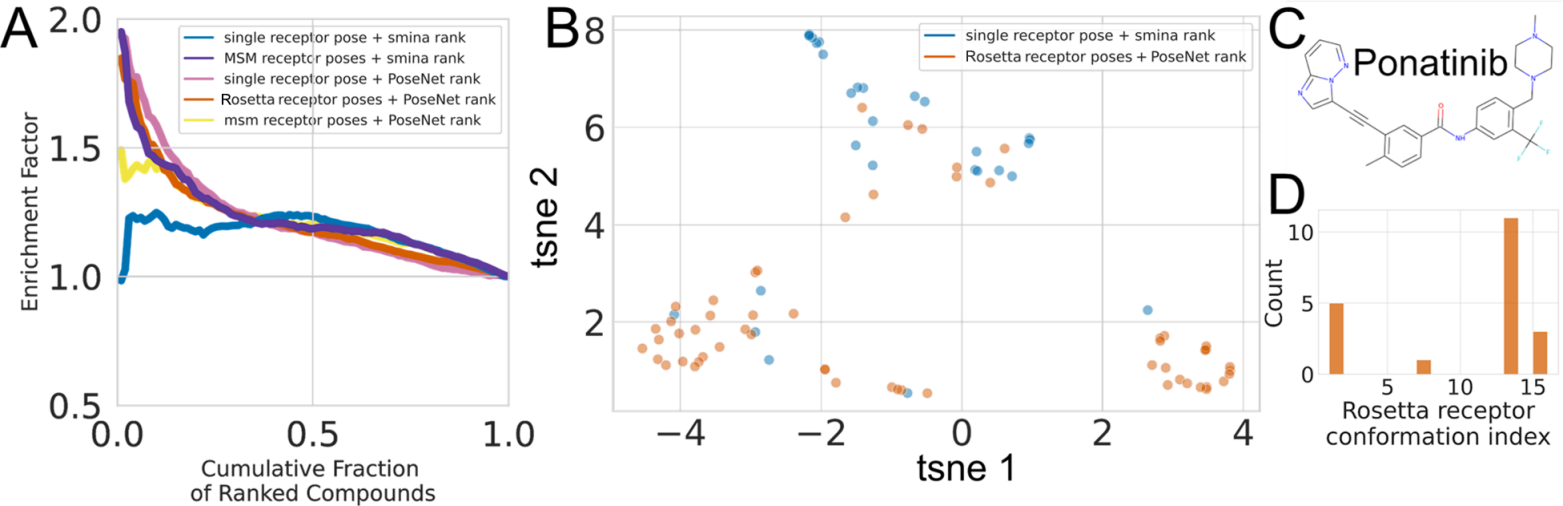
Ensemble docking enriched known Abl inhibitors compared to single receptor conformations. A) Enrichment factors for established Abl kinase inhibitors and confirmed nonbinders and random compounds. Colors represent different combinations of ensembles and scoring algorithms. Compounds are ranked from best to worst according to the score for each method, i.e., the value 0.5 on the *x*-axis represents the top-scoring 50% of compounds for each of the methods. B) Clustering by the ECFP4 fingerprint of active compounds selected by docking to a single conformation (blue) or an ensemble (orange). C) The structure of ponatinib, a known Abl1 inhibitor. Compounds in the lower right cluster of panel B are ponatinib analogs. D) The distribution of Rosetta receptor conformations for ANPR hits. Conformation 1 corresponds to the crystal structure.

Docking compounds to an ensemble of MSM receptor conformations dramatically improved early enrichment of active compounds compared to docking to a single receptor conformation when we ranked compounds by smina score (Figure 6A, purple vs blue). We observed similar enrichment factors when we ranked compounds docked to an ensemble of Rosetta-generated receptor conformations using ANPR (Figure 6A, red). While these enrichment factors are near-indistinguishable, our Rosetta ensembles were generated at a fraction of the computational cost compared to MSM ensembles. Strikingly, early enrichment remained similarly elevated when we ranked compounds docked to a *single* receptor conformation using ANPR (Figure 6A, magenta). Note that the ANPR model was trained on a cross-docked data set, which led the model to recognize distinct but valid poses of compounds corresponding to diverse receptor conformations. ANPR in combination with the MSM ensemble did not achieve the same enrichment factors (Figure 6A, yellow), likely because the MSM receptor conformations are too distinct from the Rosetta-generated conformations in the training set. Thus, docking against an ensemble of conformations can lead to early enrichment of active compounds, which can be retained using even a single receptor conformation with the ANPR model.

We then compared the chemical diversity of active top compounds docked to single receptor conformations to those using the Rosetta ensemble. We selected the 1% top-ranked compounds for each method. These 90 compounds would approximately fill a 96-well plate used for experimental validation in a vHTS screen. We then selected all active compounds among the top 1% for the single receptor conformation/smina ranking (31 compounds) and the Rosetta-ensemble/ANPR (59 compounds) method. We excluded two compounds that ranked within the top 90 in both methods, since we are primarily interested in examining their differences. A t-SNE^66^ projection of a principal components analysis computed from their ECFP4 fingerprints suggests that the Rosetta ensemble is slightly more diverse compared to the single conformation compounds (Figure 6B). Toward the lower left corner of the t-SNE projection, smina uncovers exceedingly fewer compounds. In the lower right corner, the first t-SNE coordinate separates a cluster of compounds at “tsne 1” > 2 that were enriched nearly exclusively in the ensemble. These compounds are all analogs of Ponatinib (Figure 6C), notably sharing the ethynyl linker to a imidazol-1,2-pyridazine-like hinge region of the compound. ANPR identified the most hits in this cluster against receptor conformations 1 and 16 (Figure 6D). Altogether, two out of 20 hits in this cluster were identified from docking to a single receptor (using smina, blue circles). Among all top 1% of compounds in both docking methods, only two Ponatinib analogs were identified using smina ranking.

## 5 Conclusion

In this study, we describe ANPR, a graph-based convolutional neural network trained to identify high-quality crystal-like poses from docking. Importantly, and in contrast to previous work, we use ligand conformations prepared without knowledge of the ligand conformation in the PDB structure, which is representative of use cases encountered in virtual screening applications. Using the curated data set PDBbind 2019, we demonstrate that our method is capable of improving upon enrichment and ranking metrics for pose tasks compared to the baseline established by the physics-based scoring function vina, as implemented in the open-source docking software Smina. An important aspect of pose prediction in practical use cases for drug discovery campaigns is identifying a biologically relevant and binding-competent receptor conformation. Where there is limited crystallographic information available, such as only a single crystal structure or only a homology model for a target of interest, this can be particularly challenging. We introduce a simple Rosetta-based protocol for focused conformational sampling and demonstrate that docking to small receptor ensembles can improve pose ranking metrics. The protocol is competitive in identifying active compounds for a given target compared to ensembles generated with more computationally intensive sampling techniques, such as Markov state models derived from molecular dynamics simulations. We have integrated our sampling and ANPR reranking approach with vHTS models to improve hit identification in drug discovery.^59^

## 6 Data and Software Availability

We provide input scripts and parameters for our Rosetta protocol in the Supporting Information. Our FlexPDBBind v2019 data set of sampled ensembles for PDBBind structures and our analyses scripts are freely accessible and available for download from http://atomwise.com/flexpdbbind2019. We detailed minor modifications to the Smina software (https://sourceforge.net/projects/smina/, commit ca9dcb) in the Supporting Information. AtomNet PoseRanker is proprietary software built on open-source tools Python and PyTorch. The section “AtomNet PoseRanker” provides details for reimplementation of the MPNN underlying AtomNet PoseRanker. Note that our major findings, improved pose rankings from training on a dynamic ensemble and internalization of receptor conformational flexibility, should be similarly detected by high performance MPNNs like ANPR.

## Supporting Information Available

Rosetta protocols for ensemble sampling, Supporting Table 1: AtomNet PoseRanker model hyperparameters, and software code modifications to Smina (PDF)
