# Supplementary material for: AtomNet PoseRanker: Enriching Ligand Pose Quality for Dynamic Proteins in Virtual High-Throughput Screens
Source: J Chem Inf Model. 2022 Mar 2;62(5):1178–89. doi: 10.1021/acs.jcim.1c01250 (PMC8924924; doi:10.1021/acs.jcim.1c01250)
Supplement: Supplementary file 1 — ci1c01250_si_001.pdf [file ci1c01250_si_001.pdf]

Supporting Information for:

AtomNet PoseRanker: Enriching Ligand Pose Quality for Dynamic Proteins in Virtual High Throughput Screens

Kate A. Stafford[1], Brandon M. Anderson[1], Jon Sorenson[1], and Henry van den Bedem[1,2,*]

Atomwise, Inc., 717 Market Street, Suite 800, San Francisco, CA 94103, USA.
Department of Bioengineering and Therapeutic Sciences, University of California, San Francisco, San Francisco, CA 94158, USA.

Rosetta Protocols

```
<ROSETTASCRIPTS>
        <SCOREFXNS>
            <ScoreFunction name="s3" weights="score3"/>
            <ScoreFunction name="s4" weights="score4_smooth_cart"/>
            <ScoreFunction name="fa" weights="ref2015_cart" >
                <Reweight scoretype="metalbinding_constraint" weight="1.0" />
            </ScoreFunction>
            <ScoreFunction name="r15" weights="ref2015" >
                <Reweight scoretype="pro_close" weight="0.0" />
            </ScoreFunction>
            <ScoreFunction name="cen" weights="cen_std"/>
        </SCOREFXNS>

        <MOVERS>
            <SetupMetalsMover name="metals" />
            <Hybridize name="hybridize" batch="1" stage1_increase_cycles="1.0"
stage2_increase_cycles="1.0" stage1_scorefxn="s3" stage2_scorefxn="s4"
fa_scorefxn="fa" fragprob_stage2="0.0" add_hetatm="1"
hetatm_to_protein_cst_weight="1.0" hetatm_cst_weight="1.0" stage2_temperature="0.5">
                <Template pdb="test_with_lig.pdb" cst_file="AUTO" weight="1.000" />

            </Hybridize>
            <FastRelax name="relax" scorefxn="fa" />
        </MOVERS>
        <PROTOCOLS>
            <Add mover="metals" />
            <Add mover="hybridize"/>
        </PROTOCOLS>
        <OUTPUT scorefxn="r15" />
    </ROSETTASCRIPTS>
```

Table S1. AtomNet PoseRanker model hyperparameters

Parameter description	Value
Minibatch size	64
Optimizer	adam
Learning rate	0.001
Activation function	LeakyReLU
Radial thresholds	10Å (l→l, r→l), 5Å (l→r)
Radial filter resolution (δ_ℓ)	0.15625
Atom dropout	0.15
Edge dropout	0.15
Atom embedding initial embedding dimension	32
Message passing output filters	256
Atom update hidden layer sizes	256
Readout layer hidden layer sizes	512

We trained the model by sampling a 1:1 ratio of positive to negative examples for a total of 1 million iterations, sufficient to ensure convergence for all models.

Modifications to smina

We exposed the number of Monte Carlo steps as a user parameter by adding the following changes to the file src/main/main.cpp:

Line 67:

```
double steps_fraction;
int mc_steps;
```

Line 76:

```
steps_fraction(1), score_only(false), randomize_only(false), local_only(false),
dominimize(false), include_atom_info(false), mc_steps(-1)
```

Line 418:

```
par.mc.num_steps = unsigned(par.mc.num_steps * settings.steps_fraction);
if (settings.mc_steps > -1)  // if present, --mc_steps overrides --steps_fraction
               par.mc.num_steps = (unsigned) settings.mc_steps;
```

Line 994:

```
("steps_fraction", value<double>(&settings.steps_fraction)->default_value(1.0),
                 "fraction of mc steps to perform (i.e. 1 is normal length)")
("mc_steps", value<int>(&settings.mc_steps)->default_value(-1),
                 "number of mc steps to take (if >= 0 then overrides steps_fraction)")
```
